# Indexing of Speckle Tracking Longitudinal Strain of Right Ventricle to Body Surface Area Does Not Improve Its Efficiency in Diagnosis and Mortality Risk Stratification in Patients with Acute Pulmonary Embolism

**DOI:** 10.3390/healthcare11111629

**Published:** 2023-06-02

**Authors:** Jerzy Wiliński, Anna Skwarek, Radosław Borek, Michał Medygrał, Iwona Chrzan, Marta Lechowicz-Wilińska, Ositadima Chukwu

**Affiliations:** 1 Department of Internal Medicine with Cardiology Subdivision, Blessed Marta Wiecka District Hospital, 32-700 Bochnia, Poland; skwarekanna88@gmail.com (A.S.); underhill4@gmail.com (R.B.); m.medygral@gmail.com (M.M.); 2 Center for Invasive Cardiology, Electrotherapy and Angiology, 33-300 Nowy Sącz, Poland; i.chrzan@intercard.net.pl; 3 Department of General, Plastic and Reconstructive Surgery, 5th Military Clinical Hospital with Polyclinic, 30-901 Krakow, Poland; gniewko3@gmail.com; 4 Department of Urology and Urological Oncology, Pomeranian Medical University, 71-899 Szczecin, Poland; stdmchkw@gmail.com

**Keywords:** pulmonary embolism, echocardiography, prognosis, myocardial strain, right ventricle, body surface area

## Abstract

Background: Acute pulmonary embolism (PE) is associated with a serious mortality rate. Thus, the rapid diagnosis and identification of patients at high risk of death is pivotal. The search for echocardiographic parameters for this purpose continues. Recent publications reveal correlations between myocardial longitudinal strain (LS) and body surface area (BSA). The aim of the study was to evaluate the usefulness of indexing the right ventricular (RV) speckle tracking LS to BSA in detecting PE and stratifying the risk of 30-day all-cause mortality. Methods: the prospective cross-sectional observational study group consisted of 167 consecutive patients (76 men, 45.5%) aged 69.5 ± 15.3 years, and they were referred for computed tomography pulmonary angiography. Patients underwent a transthoracic echocardiographic examination within 24 h of admission to the hospital ward. RVLS and their derivatives indexed to BSA were included in the analysis. Results: PE was confirmed in 88 patients, while 79 patients had no radiological features of PE. The only echocardiographic parameters that differed between subgroups were pulmonary flow acceleration (Act), McConnell’s sign, LS of the middle segment of the RV free wall, and its derivative indexed to BSA. During the 30-day follow-up of a subgroup of subjects with PE, 12 patients died. The mortality predictors with increasing prediction value included a RV free wall mid-segment LS (cut-off value: −21%, Area Under the Curve—AUC 0.6, *p* = 0.02) and its derivative indexed to BSA (−14 %/m^2^, AUC 0.62, *p* = 0.003), body mass index (24.7 kg/m^2^, AUC 0.63, *p* = 0.002), D-dimer serum concentration (3559 pg/mL, AUC 0.66, *p* < 0.001), Act (67 ms, AUC 0.67, *p* < 0.001), septal basal LS (−15%, AUC 0.68, *p* = 0.02), RV free wall basal segment LS (−14%, AUC 0.7, *p* = 0.015), age (66 years, AUC 0.74, *p* = 0.004), NT-proBNP (1120 pg/mL, AUC 0.75, *p* = 0.01), troponin T (66 ng/mL, AUC 0.78, *p* = 0.005), and the complex score of the Pulmonary Embolism Severity Index (AUC 0.88, *p* < 0.001). Conclusions: indexing of RVLS to BSA does not improve its prognostic value in patients with acute PE.

## 1. Introduction

Venous thromboembolism with its clinical manifestations of deep vein thrombosis and pulmonary embolism (PE) is globally the third most frequent acute cardiovascular syndrome following myocardial infarctions and strokes [1]. The disorder has been showing a steady upward trend in recent years with an annual incidence between 100 and 200 per 100,000 adults [2]. Venous thromboembolic disease with PE has a broad array of complications including the disability related to subsequent chronic thromboembolic pulmonary hypertension and impaired quality of life [3,4]. Moreover, acute PE is associated with a serious mortality rate beginning from 8% in early diagnosed and treated patients, to 30% in those untreated [5]. Thus, the rapid diagnosis and identification of individuals with acute PE at high risk of death is pivotal. Echocardiography appears to be an underestimated tool.

Transthoracic echocardiography (TTE), according to the recent versions of the guidelines for the diagnosis and management of acute PE of the European Society of Cardiology (ESC), is not a mandatory step of the routine diagnostics’ path in hemodynamically stable patients with suspected and diagnosed PE [1,6]. As the short-term prognosis in acute PE is mainly conditioned by the hemodynamic status, the dysfunction of the right ventricle (RV) detected in TTE is associated with an increased risk of short-term mortality in normotensive subjects as well [7]. Additionally, some echocardiographic findings may affect initially normotensive patients with PE and move them to the group of patients with PE of intermediate-risk, while closely monitoring heart function might help to unveil hemodynamic decompensation and identify candidates for rescue reperfusion therapy [8]. The search for TTE parameters of RV dysfunction with high predictive values continues.

One of the latest TTE techniques used to trace RV dysfunction in PE is the assessment of RV speckle tracking longitudinal strain (LS) [9]. LS describes the deformation of the segments of the cardiac chambers’ walls from a relaxed to a contracted condition. Its result is expressed as a percentage with negative values in viable myocardium when it concerns the systole. The studies on peak systolic LS of RV in acute PE, the phenomenon assessed in scientific research, have used different approaches to right ventricular longitudinal strain (RVLS) appraisal and yielded different results but what they have in common is the application of the crude values of RVLS in statistical analyses, irrespective of the body size of patients [10,11]. As recent studies on LS of the left ventricle (LV) have shown, the values of LS are correlated with body surface area (BSA) in healthy adults and children [12,13,14]. The association between RVLS and BSA is unspecified [15]. The question arises if including indexation of RVLS might increase the prognostic value of RVLS in the detection of acute PE and in prognosing a short-term outcome in PE patients.

The aim of the study is to evaluate the usefulness of indexing RVLS to BSA in detecting acute PE and stratifying the risk of death within a 30-day observation after acute PE.

## 2. Material and Methods

### 2.1. Methodology

This was a prospective cross-sectional observational single-center study. Consecutive patients of the Internal Medicine Department or its Special Care Cardiac Unit with a high clinical probability of PE were included in the study group. All of them were referred for computed tomography pulmonary angiography (CTPA). The treatment regimen followed the guidelines on PE management of ESC and was described thoroughly as conveyed previously [1,6,16].

The exclusion criteria covered echocardiograms of inadequate quality, recurrent PE or chronic thromboembolic pulmonary hypertension, severe valvular defects and tricuspid valve replacements, as well as contraindications to CTPA.

Management of the patients on the day of admission to the ward included measuring the following laboratory parameters with i.a. serum concentrations of troponin T, N-terminal pro B-type Natriuretic Peptide (NT-proBNP), and D-dimer with laboratory methods as formerly summarized [16].

**Figure 1:**
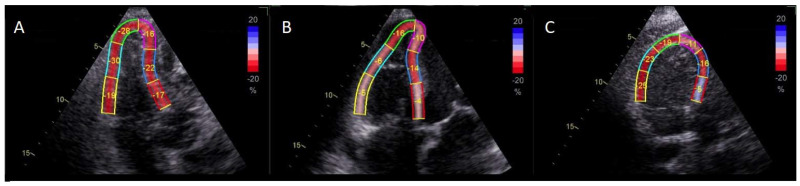
Exemplary results of the measurement of right ventricular peak systolic strain by two-dimensional speckle-tracking in a healthy patient without acute pulmonary embolism (**A**), a patient with high-risk pulmonary embolism (**B**), and a patient without pulmonary embolism but with coronary artery disease after multiple coronary interventions with hypokinetic interventricular septum (**C**).

TTEs were performed within the first day after admission to the wards by one experienced sonographer cardiologist (J.W.). Commercially available echocardiographic systems of Vivid S60 N or Vivid S6 (General Electric Company, Boston, Massachusetts, United States of America) were used. All TTEs were executed according to a predefined protocol [16,17]. The measurements were made based on the current guidelines of the European Association of Cardiovascular Imaging (EACVI) with real-time electrocardiographic recording in order to precisely define the phases of the heart cycle [18]. The estimation of RVLS by two-dimensional speckle-tracking echocardiography was performed within six segments of RV at the same time in the apical four-chamber view as recommended (Figure 1). These six segments-three RV free walls (basal, mid, and apical) and three septal ones (basal inferoseptum, mid inferoseptum, apical septum) were analyzed separately. Additionally, the average value of 3 RV free wall (RVFW) segments (RVFWLS) and the average value of the strain of all 6 RV segments-RV global LS (RVGLS) were also included in the analysis [19]. The measurements of LS were performed only by the aforementioned cardiologist.

The study endpoint was at 30-day overall mortality. Data collection during the follow-up of the study was described in detail in an earlier publication [16].

The study protocol was approved by the Bioethics Committee of the Regional Medical Chamber in Tarnow, Poland (No. 3/0177/2019). The study was performed in concordance with ethical principles of clinical research based on the Declaration of Helsinki.

### 2.2. Statistical Analysis

Statistical analysis was performed with the R Project for Statistical Computing version 4.2.1 (The R Foundation for Statistical Computing, Free Software Foundation Inc., Vienna, Austria). The Shapiro–Wilk test was applied and it disproved the normality of distribution.

Subsequently, quantitative variables are expressed as the median with an interquartile range (IQR), and the Mann–Whitney U-test was used for their comparisons. Qualitative variables are expressed as numbers (percentage) and the Fisher test or Chi square test was utilized for their comparisons when adequate. Logistic regression was used to identify predictors of PE in the whole study population and the 30-day mortality in the subgroup of patients with PE. Only single-predictor logistic regression models were utilized. The Youden index was used to calculate optimal cut-off values. Standard receiver–operating characteristic (ROC) analysis was performed, and the area under the curve (AUC), sensitivity, specificity, and corresponding 95% confidence interval (CI) were calculated. The two-sided *p*-values < 0.05 were considered statistically significant.

## 3. Results

### 3.1. Course of the Study

The study comprised 194 consecutive patients. A total of twenty-two patients had echocardiograms of poor quality. Additionally, five subjects had nondiagnostic CTPA. In effect, 167 individuals were eligible to be enrolled in the study. The baseline characteristics and biochemical parameters of these participants are presented in Table 1.

A total of 88 patients had confirmed PE: 39 subjects had central PE with thrombi in the main trunk of the pulmonary artery and/or in the right or left main pulmonary artery (44%), whereas 49 individuals (56%) had peripheral PE where segmental or subsegmental arteries were affected. Within this subgroup, 5 patients were classified with high-risk PE, 24 with intermediate-high risk, 34 with intermediate-low risk, and 25 with low-risk PE.

The subjects with PE had a higher body mass index, were less often presented with coronary artery disease and chronic heart failure, and they had elevated D-dimer serum concentration compared to individuals without PE (Table 1).

During a 30-day follow-up, some patients of the PE group passed away. A total of three subjects died due to PE, which in effect caused refractory RV failure. Four subjects required thrombolysis (they received systemic thrombolysis with alteplase) within 24 h of admission to the ward. Of these, two died and two survived. Among the next seven individuals, PE contributed to death by aggravating other decompensated diseases: heart failure in three, pneumonia in two, kidney failure in one, and disseminated neoplastic disease in one. No rescue thrombolysis was needed within the observational period.

The patients who died in the follow-up compared to the survivors were older, and had a higher score in the Pulmonary Embolism Severity Index (PESI) as well as increased troponin T and NT-proBNP serum concentrations (Table 1).

### 3.2. Echocardiographic Parameters

Patients with PE had higher values of basal right ventricular end-diastolic diameter measured in the transverse view (RVTD) and decreased values of pulmonary artery acceleration time (Act), whereas they presented more frequently with the McConnell sign compared to individuals with no signs of PE in CTPA (Table 2). In the analysis of the RV strain, the subjects with PE had lower values of LS in the RVFW mid-segment, and its derivative indexed to BSA (Table 3).

The deceased study participants, compared to the survivors, had a diminished tricuspid annular plane systolic excursion (TAPSE) and decreased non-indexed LS of both basal segments of RV: the basal free wall and basal inferoseptal ones (Table 2 and Table 3).

### 3.3. Analysis of Predictors of 30-Day All-Cause Mortality

The ROC analysis revealed 11 predictors of a fatal outcome: age, body mass index, PESI score, concentrations of D-dimer, troponin T, NT-proBNP, unindexed LS of RVFW basal segment, unindexed LS of the basal inferoseptal segment, and unindexed LS of RVFW mid-segment and its derivative indexed to BSA and Act (Table 4).

## 4. Discussion

The systolic function of RV has an important role in the prediction of unfavorable outcomes in a broad range of cardiovascular disorders. Unfortunately, complex RV geometry forecloses the determination of a single universal parameter that could reliably reflect the size and function of this heart chamber [1].

The advantages of RVLS measuring regional myocardial deformation cover high reproducibility, evaluation of the mechanical function of the full RV wall thickness, relative load independency, angle-independency, high availability, low costs, short scan duration, and the lack of need for advanced training. The disadvantages include disagreement with regards to normal values and three- and six-segment models, a necessity of a stable heart rhythm and high temporal resolution, as well as problems with the imaging window and visualization of RVFW and the endocardial border [20]. Of note, RVLS could not be obtained in over 13% of our study participants.

RVLS has shown a predictive value in patients with pulmonary hypertension, ischemic heart disease, heart failure, cardiomyopathies, congenital heart disease, and valvular diseases [21,22,23,24]. It enables the detection of subclinical RV damage in various diseases, including cardiomyopathies, cardiac amyloidosis, cancer, and pulmonary arterial hypertension even when conventional parameters of RV systolic function are in the normal range [20,25,26,27]. Measurement of RVLS can also help to predict the outcome of certain invasive procedures. Low RVLS was associated with sustained right RV dysfunction after balloon pulmonary angioplasty in individuals with chronic thromboembolic pulmonary hypertension [28]. Similar observations come from studies on LS of LV. In optimally treated patients with ST elevation myocardial infarction dysfunction of remote myocardium assessed by LS was predicted by elevated NT-proBNP, could be independent of coronary artery disease extent and infarct size, and was associated with worse LV morphological and functional indexes when followed-up [29].

Interpretation of RVLS should be done with caution. In a recent meta-analysis on 4439 healthy subjects from 45 eligible studies devoted to defining the reference range of RVLS, the meta-regression analysis conveyed that the associations between BSA and RVGLS, and BSA and RVFWLS did not reach statistical significance. However, the strongest associations from the group of variables include RV fractional area change and RV systolic pressure, and LVGLS (beta coefficients from -0.46 to 1.34) concerned the vendor—not GE EchoPAC versus GE EchoPAC with a beta of 3.9 (1.73–6.07, 95% Confidence Interval, *p*<0.001) for association with a lower limit of normal RVGLS. No analyses of subgroups of different vendors are available in this paper [15]. The significance of this relation was clearly shown in the study by Lee and colleagues on 50 patients with acute PE in whom RVGLS was measured on the same set of echocardiographic images with GE EchoPAC and Siemens Medical System VVI. The RVGLS of both vendors were correlated (r = 0.793, *p* < 0.001) and they showed significant correlations with conventional echocardiographic parameters of RV systolic function and B-type natriuretic peptide serum concentration. However, only RVGLS VVI, and not RVGLS EchoPAC, showed significant correlations with cardiac biomarkers as serum creatinine kinase-MB (r = 0.367, *p* = 0.010) and troponin I concentrations (r = 0.294, *p* = 0.040) [11].

If PE patients are compared to healthy controls as seen in a study by Trivedi and colleagues, RVFWLS is a great discriminator for PE. In comparative multiple logistic regression models for PE, the model which included traditional measures of RV size and function and RVFWLS produced a powerful classifier (AUC 0.966, SE 0.013, *p* < 0.022) with significantly better performance than the model without RVFWLS [9]. Unfortunately, in real-life clinical settings, most PE-suspected individuals are multimorbid patients with chronic diseases of the heart and lungs which affect RV performance and alter RVLS [30]. Only in our study, within the subgroup of patients without PE, almost 38% had chronic heart failure and 12.7% had chronic lung disease.

Patients with acute PE tend to have reduced regional RVLS, especially in the basal and mid-segments of RVFW [10,31,32]. In the presented study results, the difference concerned only the RV mid-segment. In our opinion, the assessment of the LS of the mid-segment is more reliable and more reproducible than the LS of the basal one due to a more pronounced mechanical movement of the basal part of RVFW, which as reflected by TAPSE at times exceeds 30 mm. Of note, it was the LS of the mid-RVFW segment that was the only TTE parameter of RV systolic function that enabled the differentiation between acute cor pulmonale complicated with massive PE from its chronic form due to severe chronic obstructive pulmonary disease at an emergency department [33]. The indexation of RVLS to BSA did not bring additional benefits in PE diagnostics.

Considering prognostication, in the study by Dahhan and colleagues RVGLS and RVFWLS, apart from the Tei index, were the only TTE predictors of mortality after acute PE [34]. Similar findings come from the publication by Lee et al., in which RVGLS and RVFWLS independently predicted in-hospital vents: death, the need for additive treatments such as thrombolysis or pulmonary artery thromboembolectomy, and the need for inotropes due to unstable vital signs [35]. RVFWLS was also a predictor of mortality in patients after acute PE in a longer 12-month follow-up [36].

Our study shows that a coarse analysis of LS based on a global or free wall assessment is not always sufficient to reveal differences among certain groups of patients. The LS of the RVFW basal and mid-segments and basal inferoseptum segments were key in our survey. Similar findings come from the paper by Vitarelli et al., where TTE was extended by a three-dimensional technique (3D). In multivariate analysis, RV systolic pressure, 3D RV ejection fraction, and the LS od RVFW mid-segment were independently associated with adverse outcomes [37]. Interestingly, as the septum is anatomically related to the LV, there is currently no agreement over how to evaluate RV function using LS [38]. Septal LS might also be affected by LV disorders and mislead the RV systolic function assessment when septal LS is considered. LV dysfunction can also complicate PE and secondarily affect RV dysfunction from this perspective. In the paper by Plats and colleagues, regional RVLS was markedly reduced in PE subjects compared to the healthy control group in all regions of the RVFW and in the mid and basal septum [10]. LS of the basal septal segment was significantly decreased in non-survivors in our study.

The only non-LS TTE parameter with a predictive value in our survey is Act. Its association with 30-day mortality after acute PE was observed in a prospective blinded study and in our former calculations [16,39]. The results of our analysis show that TTE parameters as stand-alone predictors of short-term mortality are poor discriminators with an AUC 0.6–0.7 at the level of age and BMI but inferior to the fair discriminator NT-proBNP and troponin T with an AUC of 0.75 and 0.78, respectively, as well as inferior to a good discriminator PESI with an AUC of 0.88. PESI is a validated and recommended tool in ESC guidelines that incorporates multiple variables of comorbidities, age, and clinical and mental status [1]. Integration of echocardiographic parameters and cardiac markers could be an alternative or supplementary aspect to clinical scores, especially when cut-off values are individualized and related, e.g., to patients’ ages [40]. Importantly, other diseases might influence the clinical and biochemical variables. Within our study, subjects with clinical suspicion of acute PE but with no PE signs upon CTPA had significantly more often coronary artery and congestive heart failure.

In summary, despite mounting evidence that supports the evaluation of RVLS in the majority of patients with cardiovascular diseases, it has not yet become part of a routine TTE examination in the majority of echocardiographic laboratories [20]. The addition of RVLS analysis to existing parameters of RV size and function and clinical data might significantly improve sensitivity and specificity for the diagnosis of PE and may play a role in the diagnosis, guiding therapy and predicting outcomes [9].

## 5. Conclusions

Right ventricular strain assessed by two-dimensional speckle-tracking echocardiography differs between patients with and without acute pulmonary embolism and between survivors and non-survivors of acute pulmonary embolism. Those differences concern individual segments of the RV free wall and interventricular septum. The addition of RVLS analysis to routine appraisal of RV in echocardiography might improve sensitivity and specificity for the diagnosis of PE and may play a role in predicting adverse outcomes. The indexing of the RVLS to the BSA does not improve its effectiveness in predicting PE and 30-day all-cause mortality.

## 6. Study Limitation

The study has a low number of participants. Echocardiograms were not repeated, and, thus, variability of echocardiographic parameters could not be assessed. The prognostic value of biomarkers with different cut-off values was not investigated.

## Figures and Tables

**Table 1:** Clinical characteristics and selected biochemical parameters of the study participants: all patients, subgroups of individuals with and without acute pulmonary embolism, deceased subjects, and survivors within 30-days of observation.

	All Subjects (*n* = 167)	Patients with No PE (*n* = 79)	Patients with PE (*n* = 88)	*p*	Survivors (*n* = 76)	Non-Survivors (*n* = 12)	*p*
Gender [male]	76 (45.51%)	32 (40.51%)	44 (50%)	0.36	41 (53.95%)	3 (25%)	0.12
Age [years]	69 (59.50–79.08)	70 (61.21–78.51)	69 (58.14–79.37)	0.95	66 (56.75–77.50)	74.5 (69–89.25)	0.01
BMI [kg/m^2^]	27.55 (23.84–31.08)	26.61 (22.39–29.47)	28.22 (25.97–31.25)	0.02	28.34 (26.07–31.25)	27.3 (23.93–30.99)	0.46
Arterial hypertension	102 (61.08%)	50 (63.29%)	52 (59.09%)	0.64	46 (60.53%)	6 (50%)	0.49
Hyperlipidemia	72 (43.11%)	39 (49.37%)	33 (37.5%)	0.08	29 (38.16%)	4 (33.33%)	1
Diabetes mellitus	38 (22.75%)	18 (22.78%)	20 (22.73%)	0.72	17 (22.37%)	3 (25.00%)	1
Coronary artery disease	44 (26.35%)	28 (35.44%)	16 (18.18%)	0.01	15 (19.74%)	1 (8.33%)	0.69
Chronic heart failure	51 (30.54%)	30 (37.97%)	21 (23.86%)	0.01	18 (23.68%)	3 (25%)	0.76
Atrial fibrillation (present or prior)	21 (12.57%)	10 (12.66%)	11 (12.50%)	0.28	9 (11.84%)	2 (16.67%)	0.64
Chronic lung disease	17 (10.18%)	10 (12.66%)	7 (7.95%)	0.09	6 (7.89%)	1 (8.33%)	1
Active malignancy	38 (22.75%)	19 (24.05%)	19 (21.59%)	0.67	16 (21.05%)	3 (25.00%)	0.69
Acute infection	65 (38.92%)	31 (39.24%)	34 (38.64%)	0.29	28 (36.84%)	6 (50.00%)	0.38
PESI [pts]	94 (79–116)	94 (79–117)	94 (78–115)	0.42	90 (70–107)	136 (113–173)	<0.001
Troponin T [pg/mL]	21.51 (11.53–45.75)	21.74 (13.14–48.81)	20.87 (10.73–45.61)	0.56	18.84 (9.20–44.57)	70.29 (28.62–136.08)	0.01
NT-proBNP [pg/mL]	811.00 (196.00–3119.00)	878.00 (229.00–3950.00)	627.50 (152.00–2878.00)	0.30	548.00 (142.00–2631.00)	2984.00 (1711.00–7791.00)	0.02
D-dimer [ng/mL]	3697.00 (1990.00–6998.00)	2898.00 (1721.00–4805.00)	4792.00 (2657.00–7801.00)	<0.001	4792.00 (2348.00–7801.00)	4737.00 (4280.00–7499.00)	0.62
Creatinine clearance [mL/min]	82.00 (62.12–104.47)	81.55 (60.25–102.60)	83.3 (67.07–106.12)	0.47	84.40 (69.35–104.97)	70.65 (42.50–132.15)	0.54

Abbreviations: PE—pulmonary embolism, BMI—Body Mass Index, PESI—Pulmonary Embolism Severity Index, and NT-proBNP—N-terminal pro-B-type natriuretic peptide.

**Table 2:** Selected echocardiographic parameters.

	Patients with No PE (*n* = 79)	Patients with PE (*n* = 88)	*p*	Survivors (*n* = 76)	Non-Survivors (*n* = 12)	*p*
RVTD [mm]	38.25 (35.17–42.43)	40.11 (37.06–43.16)	0.048	41.35 (37.49–43.25)	38.11 (35.75–42.23)	0.30
LVTD [mm]	43.65 (38.22–49.85)	44.33 (39.05–47.09)	0.98	44.21 (40.16–48.68)	40.29 (35.51–44.09)	0.06
RVTD/LVTD	0.88 (0.77–1.03)	0.93 (0.84–1.05)	0.11	0.93 (0.83–1.04)	1 (0.89–1.08)	0.38
Act [ms]	95.04 (74.11–111.82)	72.41 (57.75–96.22)	<0.001	74.16 (58.75–98.88)	59.07 (47.50–72.51)	0.07
TRPG [mm Hg]	2.72 (2.30–3.16)	2.74 (2.31–3.13)	0.86	2.75 (2.36–3.11)	2.72 (2.11–3.25)	0.94
TAPSE [mm]	22.14 (17.09–25.76)	21.45 (17.55–24.25)	0.78	22.07 (18.19–25.25)	18.50 (15.75–20.04)	0.06
TASV TDI [cm/s]	16.33 (12.11–20.44)	15.20 (13.07–19.23)	0.87	15.23 (13.17–18.51)	19.04 (14.52–20.55)	0.14
McConnell sign	1 (1.27%)	10 (11.36%)	0.01	9 (11.84%)	1 (8.33%)	1
LVEF [%]	56.31 (48.52–65.04)	56.15 (50.41–63.17)	0.93	55.52 (50.07–63.44)	56.22 (48.54–62.56)	0.95
RV FAC [%]	46.58 (37.14–51.36)	40.26 (31.03–48.33)	0.31	40.26 (29.69–49.17)	39.13 (37.16–41.10)	0.81
LVGLS [%]	16.90 (13.31–19.75)	17.20 (14.02–19.85)	0.49	17.21 (14.52–19.65)	16.61 (13.07–20.95)	0.93
RVOT PLAX [mm]	30.00 (27.00–32.00)	30.00 (28.50–34.00)	0.22	31.00(28.00–34.00)	28.00 (26.00–33.00)	0.13
RVOT SAX diastolic [mm]	32.00 (28.00–35.75)	32.00 (29.00–35.00)	0.76	32.00 (29.00–36.00)	30 (29.00–32.50)	0.10
RVOT SAX systolic [mm]	20.00 (17.00–25.75)	19.50 (16.00–24.00)	0.82	20.00 (16.00–25.00)	19 (16.00–22.75)	0.53

Abbreviations: Act—pulmonary acceleration time, LVEF—left ventricular ejection fraction, LVGLS—left ventricular global longitudinal strain, LVTD—basal left ventricular end-diastolic diameter measured in measured in the transverse view, PE—pulmonary embolism, RV FAC—right ventricular fraction area change, RVOT PLAX—right ventricular outflow tract in parasternal long axis view, RVOT SAX—right ventricular outflow tract in parasternal short axis view, RVTD-basal right ventricular end-diastolic diameter measured in the transverse view, TAPSE—tricuspid annular plane systolic excursion, TASV TDI—tricuspid annulus’ peak systolic velocity measured with tissue Doppler imaging, and TRPG—tricuspid valve peak systolic gradient.

**Table 3:** Right ventricular longitudinal strain values (absolute values).

Segments	Patients with No PE (*n* = 79)	Patients with PE (*n* = 88)	*p*	Survivors (*n* = 76)	Non-Survivors (*n* = 12)	*p*
RV strain original values [%]						
RVFW basal	23.00 (16.00–25.00)	21.00 (14.00–26.00)	0.45	21.00 (16.75–27.00)	13.00 (9.50–21.25)	0.03
RVFW mid	22.00 (17.50–28.00)	20.00 (14.75–25.00)	0.03	20.00 (16.00–25.00)	15 (10.75–23.50)	0.18
RVFW apical	18.00 (12.00–25.00)	18.00 (14.00–23.25)	0.84	18.00 (14.00–23.25)	17.00 (8.00–21.25)	0.41
SEP basal	16.00 (10.00–19.00)	17.00 (11.00–21.00)	0.10	17.00 (11.75–21.00)	13.5 (8.25–16.25)	0.049
SEP mid	18.00 (13.00–21.00)	17.00 (13.75–20.50)	0.78	17.00 (14.00–22.00)	15.00 (12.50–20.00)	0.32
SEP apical	16.00 (11.50–22.00)	16.00 (13.75–22.25)	0.40	16.00 (14.00–22.00)	16.50 (6.75–23.00)	0.58
RVFW-average of 3 segments	19.67 (14.83–25.83)	18.5 (15.33–22.92)	0.43	19 (15.67–22.92)	16.67 (10.75–22.58)	0.32
RVGLS	18.50 (14.17–22.08)	17.75 (14.46–21.5)	0.64	18 (14.88–21.12)	16 (10.62–22.58)	0.23
RV strain indexed to BSA [%/m^2^]						
RVFW basal	11.52 (8.88–14.65)	11.26 (7.53–13.59)	0.13	11.49 (7.85–13.67)	7.34 (4.9–10.87)	0.08
RVFW mid	12.27 (9.02–15.76)	10.12 (7.4–12.96)	0.01	10.17 (7.71–12.92)	7.29 (5.63–13.86)	0.37
RVFW apical	10.16 (6.71–13.39)	9.39 (6.89–11.99)	0.36	9.39 (7.15–11.89)	8.65 (4.95–12.08)	0.56
SEP basal	8.36 (5.49–10.53)	8.47 (5.72–10.77)	0.53	8.53 (6.11–10.98)	7.06 (4.7–9.79)	0.23
SEP mid	9.92 (7.28–11.98)	8.56 (6.82–11.34)	0.24	8.51 (6.96–11.34)	9.02 (5.93–10.9)	0.97
SEP apical	8.65 (6.04–12.23)	8.55 (6.6–11.78)	0.96	8.55 (6.66–11.78)	9.12 (3.25–12.09)	0.77
RVFW-average of 3 segments	10.20 (7.89–14.38)	9.43 (7.51–12.03)	0.12	9.67 (7.63–11.88)	8.62 (5.86–12.37)	0.61
RVGLS	9.71 (7.71–12.59)	8.81 (7.5–11.51)	0.22	8.81 (7.61–11.51)	8.51 (5.85–11.55)	0.66

Abbreviations: PE—pulmonary embolism, RV—right ventricular, RVFW—right ventricular free wall, RVGLS—right ventricular global longitudinal strain, amd SEP—inferoseptal/septal.

**Table 4:** Receiver–operating characteristic (ROC) analysis for 30-day all-cause mortality in 88 patients with acute pulmonary embolism.

	AUC	*p*	Youden Cut-off	Sensitivity	Specificity
Age [years]	0.74 (0.61, 0.87)	0.004	66	1 (0.74–1)	0.49 (0.37–0.6)
BMI [kg/m2]	0.63 (0.54, 0.72)	0.002	24.7	0.82 (0.72–0.89)	0.44 (0.33–0.56)
PESI [pts]	0.88 (0.80, 0.96)	<0.001	100	1 (0.74–1)	0.7 (0.59–0.8)
D-dimer [pg/mL]	0.66 (0.57, 0.75)	<0.001	3559	0.7 (0.58–0.79)	0.6 (0.48–0.71)
Troponin T [ng/mL]	0.78 (0.60, 0.95)	0.005	66	0.62 (0.24–0.91)	0.88 (0.78–0.94)
NT-proBNP [pg/mL]	0.75 (0.58, 0.92)	0.01	1120	0.89 (0.52–1)	0.62 (0.5–0.73)
RVFW basal LS [%]	0.70 (0.54, 0.86)	0.02	−14	0.58 (0.28–0.85)	0.78 (0.67–0.86)
SEP basal LS [%]	0.68 (0.49, 0.86)	0.02	−15	0.5 (0.21–0.79)	0.67 (0.55–0.77)
RVFW mid LS [%]	0.60 (0.51, 0.69)	0.01	−21	0.61 (0.5–0.72)	0.54 (0.43–0.66)
RVFW mid LS/ BSA [%/m2]	0.62 (0.54, 0.71)	0.003	−14	0.84 (0.75–0.91)	0.39 (0.28–0.51)
Act [ms]	0.67 (0.59, 0.75)	<0.001	67	0.48 (0.37–0.59)	0.82 (0.72–0.9)

Abbreviations: Act—pulmonary artery acceleration time, BMI—body mass index, LS—longitudinal strain, NT-proBNP—N-terminal pro-B-type natriuretic peptide, PESI—Pulmonary Embolism Severity Index, RV—right ventricular, RVFW—right ventricular free wall, SEP—interventricular/septal, and TAPSE—tricuspid annular plane systolic excursion.

## Data Availability

The data that support the findings of this study are available from the corresponding author upon reasonable request.
